# Investigation on the regulatory T cells signature and relevant Foxp3/STAT3 axis in esophageal cancer

**DOI:** 10.1002/cam4.5194

**Published:** 2022-10-13

**Authors:** Lin Yang, Qijie Zhao, Xing Wang, Chalermchai Pilapong, Yi Li, Jun Zou, Jing Jin, Jinfeng Rong

**Affiliations:** ^1^ Department of Oncology The Second Peopleʼs Hospital of Yibin Yibin Peopleʼs Republic of China; ^2^ Department of Pharmacy, West China Hospital Sichuan University Chengdu Peopleʼs Republic of China; ^3^ Center of Excellence for Molecular Imaging (CEMI), Department of Radiologic Technology, Faculty of Associated Medical Sciences Chiang Mai University Chiang Mai Thailand; ^4^ Shichuan Nursing Vocational College Chengdu Peopleʼs Republic of China

**Keywords:** Esophageal cancer (EC), Foxp3, immune infiltration, STAT3, Tregs

## Abstract

**Background:**

Regulatory T cells (Tregs) have an important role in accelerating the immunosuppression of tumor. Tregs regulation is a hopeful strategy to improve the dismal prognosis of Esophageal cancer (EC), while its mechanisms have not yet been fully clarified.

**Methods:**

To characterize the role of Tregs in EC, we comprehensively explored its prognostic value, clinical pathology partnership, related biological functions and potential mechanisms at transcriptome level. Through the integrated analysis of GEO and TCGA datasets, we comprehensively evaluated the Tregs infiltration patterns in EC patients. The correlation between Tregs infiltration and genomic characteristics, as well as biological functions were analyzed by a variety of computational algorithms.

**Results:**

We observed that Tregs were significantly upregulated in EC and involved in various immune processes. According to TCGA and GEO transcriptional classification schemes, Tregs specific genes were observed to be highly expressed in tumor samples, as well as were closely associated with poor prognosis and worse clinical outcomes. In addition, EC patients can be stratified into high‐risk and low‐risk immune subgroups according to Tregs/macrophages infiltration level, and the results showed significant differences in tumor development, biological processes and probe gene expression pattern. The multi‐variate analysis revealed that the interaction between STAT3 and Foxp3 was a potential prognostic signature of Tregs in EC, especially the modulation effect of STAT3 on Foxp3 expression, which has not been well studied in EC. We also identified that STAT3 and Foxp3 expression presented a high accuracy in predicting Tregs infiltration level in EC patients (AUC: 0.817; 95% CI: 0.756–0.878).

**Conclusions:**

Our results revealed that Tregs have the potential to predict prognosis and tumor deterioration in EC patients. A comprehensive landscape of Tregs regulation mechanisms will help us interpret the immunosuppression of tumor microenvironment (TME) and novel strategies for EC immunotherapy.

## Graphical abstract

Regulatory T cells (Tregs) are well‐known immunosuppression factor in most human malignancies. We gave a comprehensive analysis of Tregs disorder and observed that high Tregs accumulation potentially acted as a prognostic indicator for esophageal cancer (EC) and was associated with tumor deterioration.

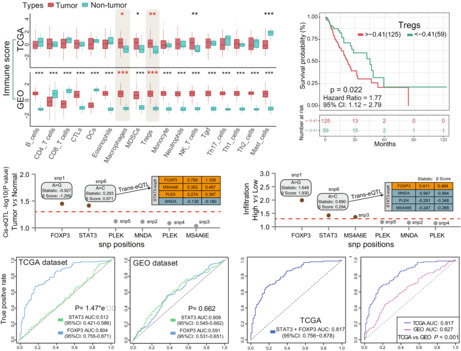

## 1 INTRODUCTION

Esophageal cancer (EC) has been regarded as the sixth highly lethal malignant tumors worldwide.^1^ Currently, the most common treatment strategies include surgical operation, chemo‐therapy and radiotherapy, but prognosis remains poor and the overall 5‐year survival rate ranges from 15% to 25%.^2^ Generally, diagnosis made at the early stages is associated with better outcomes than those made at the advanced stages.^3^ Recently, when advanced EC patients received immunotherapy, some promising effects were observed.^4^ Studies have reported that diverse immune cell populations in the tumor microenvironment (TME) of EC were associated with pro‐tumorigenic function, anti‐tumor immune response, invasion and metastasis.^5^, ^6^ Immune cells in the tumor milieu represent a mechanism that is important for systematic anti‐tumor activity and tumor immunosurveillance.^7^

Tumor immune evasion and impaired antitumor immunity are critical for tumor development and metastasis.^8^ The compositions of different TME immune cells suggested that immune cells play a crucial role in tumor progression and therapeutic response, such as Regulatory T cells (Tregs), T cells, Dendritic cells (DCs), Macrophages and Myeloid‐derived suppressor cells (MDSCs) et. al.^9^, ^10^, ^11^, ^12^ In tumor physiology, Tregs favor the suppression and exhaustion of T cells, B cells and natural killer cells (NKs), which have immunosuppressive abilities that promote immune evasion and attenuate antitumor immune response in TME.^13^, ^14^ Tregs infiltration was deemed as a prognostic indicator of EC, and higher amount of Tregs is connected with worse tumor invasion,^15^ metastasis,^16^ disease progression,^17^ and poor survival after chemotherapy.^18^ Moreover, the dual roles of Tregs are closely associated with its self‐activated molecules, such as Forkhead box P3‐positive (Foxp3^+^) and CD25, and its dysregulation may contribute to immune disorder and inflammation.^18^, ^19^ Immunotherapy and tumors treatment responses are usually accompanied by complex networks between molecules and signaling pathways. Of note, in recent, several biomarkers were proved to have the ability to predict the tumor status and therapeutic effects.^20^ Interestingly, increased recruitment of Foxp3^+^ Tregs were observed in EC.^21^ Recently, the number and function of Foxp3^+^ Tregs have been reported to be associated with STAT3 expression level,^22^, ^23^, ^24^ and STAT3 was simultaneously regarded as an potent therapeutic target for Tregs in head and neck cancer.^25^ In addition, increased Foxp3 gene expression and Foxp3^+^ Tregs accumulation were also observed in the regions near the tumor metastasis proximity site, as well as higher STAT3 expression.^26^ However, the landscape of EC with immune cells infiltration and STAT3‐related mechanisms still remained to be determined.

A deeper understanding of immune cells regulation mechanisms in esophageal tumorigenesis and TME can help us improve the diagnostic accuracy and future therapeutic effects. In this study, we explored the differentially expressed Tregs probe genes in EC and related immune signature, which is superior to the traditional study perspective. We employed two established computational algorithms to evaluate the different immune cells fraction via the signature probes expression profiles in EC patients.^27^, ^28^ In addition, Foxp3^+^ Tregs related infiltration pattern was integrated with the genomic characteristics, molecules interaction and clinical features in EC patients. As a result, we observed that Tregs were closely associated with EC development and poor prognosis, and the STAT3/Foxp3 axis potentially be a predictive signature of Tregs in EC patients.

## 2 MATERIALS AND METHODS

### 2.1 Esophageal cancer datasets and preprocessing

EC patients Transcriptome data were acquired from two independent databases: The Cancer Genome Atlas (TCGA) dataset (*n* = 195) and Gene Expression Omnibus (GEO) dataset (*n* = 545). Totally, eleven EC sample cohorts were employed in our study: TCGA‐ESCA, GSE69925, GSE45670, GSE37200, GSE37203, GSE67269, GSE20347, GSE44021, GSE39491, GSE23400, and GSE13083 (Table S1). The raw data from the GEO Affymetrix microarray data sets were firstly performed the background adjustment according to the RMA algorithm in Affy package.^29^ In the RMA, median polish algorithm was used to process oligonucleotides per transcript adjustment, quantile normalization, and final statistics. For the TCGA database, EC patient gene expression profiles (RNAseq: FPKM value) were obtained by the UCSC Xena browser (GDC_hub: gdc.xenahubs.net). To integrate the genes profiles from different platforms, we applied the unified Entrez_ID annotation for gene symbol. Different cohorts from the same test platform were summarized and batch effects were eliminated with ComBat software.^30^ Then, the redundancy of probes gene expression was checked and corrected in each cohort. Eligible baseline information like follow‐up time, vital status, pathological stages, and metastasis were downloaded from TCGA database.

TCGA‐ESCA individual genomic variations (Affymetrix SNP 6.0) from the Broad Institute platform were obtained from Copy Number Variation (CNV) analysis. GISTIC_2.0 software was used to identify the significantly amplified or deleted genomic loci. Copy number loss or gain burden at the focal and broad‐levels were evaluated in the number of genes. CHIP‐Seq data was obtain from GEO database (GSE123398), Naïve B cells. The sequence data for the expression quantitative trait locus (cis‐ and trans‐eQTL) study was filtered based on somatic mutation files, and forward stepwise conditional analysis implemented in MatrixEQTL algorithm was conducted.

### 2.2 Immune cells‐metagene definition

The predictive signature probes of selected immune cells were summarized and integrated by the sources of Ru et al.^31^ (http://cis.hku.hk/TISIDB/) and Bindea et al.^32^ The establishment of immune cell metagenes includes 24 immune categories according to different immune cell types, such as T cells, B cells, DCs, macrophages and Tregs.

### 2.3 Generation of immune cells gene signatures

To quantify immune cell dysregulation patterns in different EC patients, we established an immune cell score evaluation algorithm for individual EC samples by using above immune cells‐metagene signatures, which was defined as the Tregs signature score. The gene expression profile for each signature was firstly transformed into z‐score. Then, immune cell‐related gene signatures were calculated by principal component analysis (PCA), where component_1 was extracted and termed as the signature score. The advantage of this method is that the scores are concentrated on the set with largest block of well‐correlated (or anticorrelated) genes in set. We then defined a signature score by using a method similar to GGI^33^, ^34^ for each patient:

Immune score:∑PC1i−∑PC1j

According to Cox coefficient, *i* is the positive clusters signature score, and *j* is negative signature score.

A nomogram was constructed by integrating the Tregs score and clinical parameters, including tumor grade, site, metastasis, and clinical stage. The nomogram was used to evaluate each variable score, and by calculating the total score, we estimated the 3‐ and 5‐year survival rates. In addition, the time‐dependent ROC curve and calibration curve (Figure S1B‐D) were used to estimate the prediction ability and accuracy of the nomogram, respectively.

### 2.4 Inference of immune cells infiltration in esophageal cancer

For the EC sample's different immune cells proportions evaluation, single‐sample gene‐set enrichment analysis (ssGSEA) algorithm was utilized for immune cells infiltration analysis. For the above 24 types of immune‐cell metagenes, the relative abundance of immune cell infiltration in each sample was summarized by ssGSEA. Simultaneously, eight immune cell populations and two non‐immune cell populations were evaluated by the MCP‐counter method as previously described.^27^

### 2.5 Biological function enrichment analysis

To further understand Tregs genetic functions and its relevant network in EC, KEGG and GO databases were employed for selected markers biological function enrichment analysis. The analysis mainly includes three parts: biological process (BP), molecular function (MF) and cellular components (CC). For multiple comparison, the *p* value was adjusted by the False‐Discovery Rate (FDR) algorithm. The ClusterProfiler software was used for all enrichment analysis.^35^ In current study, according to the Tregs/macrophages infiltration clustering, entire TCGA EC patients were stratified into high‐ and low‐infiltration cohorts. Differences in immune cell‐related biological function were investigated by two cohorts comparison. The criterion screening was chosen *p* value <0.05. The enrichment results of Tregs‐relevant signature clusters were represented by enrichplot software. The enrichplot is a development package with implemented visualization approaches to interpret biological function results, which is available from the github repository.

### 2.6 Network analysis

To identify the genomic association between Tregs‐metagene and STAT3, the gene symbols were used as parameter and queried in the STRING database.^36^ The STRING has the ability to build complex interaction networks by retrieving interacting Genes/Proteins. Based on co‐expression, experimentally‐interaction homologies and database‐annotated information, we summarized the combined scores for further analysis.

### 2.7 Statistical analysis

All analyses were performed by using R (https://www.r‐project.org/) with several publicly available packages and SPSS software (version 24.0). Different gene profile correlation coefficients were evaluated by Spearman and distance correlation analysis. For two groups comparison, unpaired Student *t* test was employed for statistical difference analysis. Kruskal‐Wallis test was used for comparative analysis of statistical differences among multiple cohorts.^37^ In terms of immune‐cell relevant scores and survival‐related correlations, the survminer software was used to determine the cohort cut‐off point and stratify the subgroups. In order to ascertain the maximum rank statistic, “Surv‐cutpoint” function iteratively checked all possible cohort cut points, respectively. The appropriate separation parameter was adopted to dichotomize immune‐cell relevant scores, where the cohorts were stratified into low and high subgroups based on the most adapted log‐rank statistics. This approach has the potential to reduce the batch effect of calculation. Kaplan–Meier curve was applied for survival analysis, and statistical differences were measured by log‐rank test. Multivariate prognostic analysis for Tregs immune‐score was calculated and visualized by the forestplot software. The univariate Cox regression model was used to evaluate the hazard ratios (HR) for each prognostic cohorts. The independent prognostic factors were estimated with multivariate Cox regression model. The diagnostic accuracy of STAT3, Tregs‐relevant markers, and the combination of different parameters were evaluated by pROC software.^38^ The pROC has the ability to calculate the area under the curve (AUC) and confidence intervals by using receiver operating characteristic (ROC) curve. For the different ROC curves comparison, likelihood test was used to calculate statistical difference. *p*‐value <0.05 is considered statistically significant.

## 3 RESULTS

### 3.1 Identification of Tregs expansion status in esophageal cancer

**FIGURE 1:**
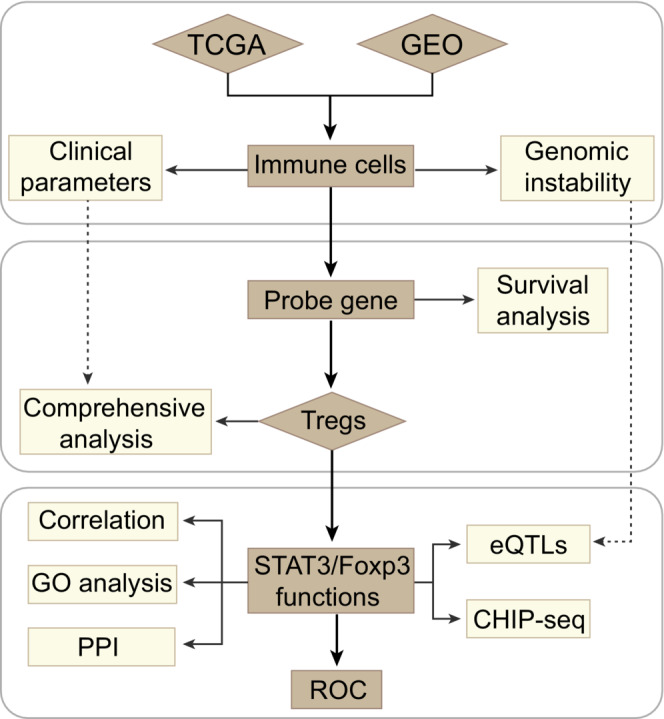
Flowchart of the study. Eleven available EC datasets with 740 tumor and normal samples were employed from the TCGA and GEO database. We evaluated the EC related immune cells signature in two independent platforms and focused on Tregs related characteristics. Further, we integrated the patient clinical parameters, genomic alteration, STAT3/Foxp3 functions, and biological processes to exploring the Tregs characteristics in EC. EC, Esophageal cancer; GEO, Gene Expression Omnibus; TCGA, The Cancer Genome Atlas

**FIGURE 2:**
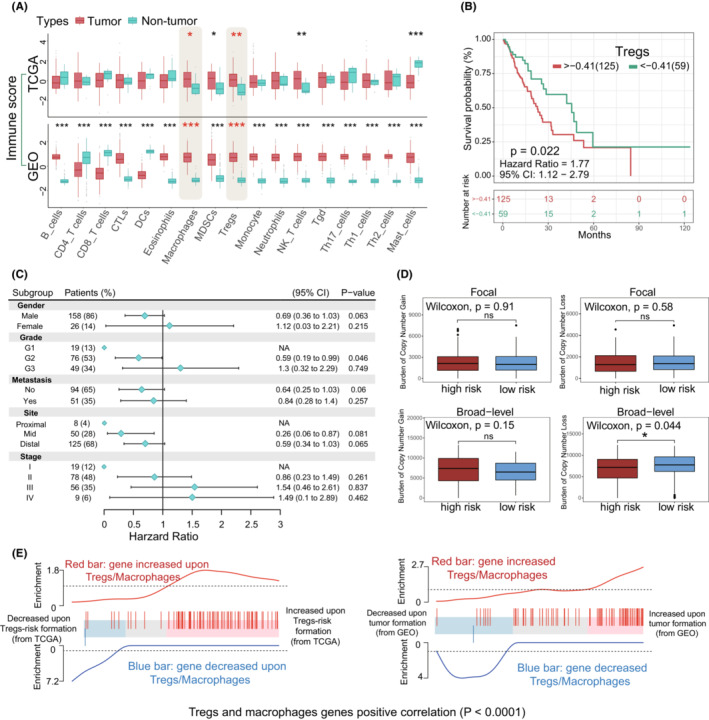
Landscape of the immune cell subtypes and Tregs characteristics in esophageal cancer. (A) 17 distinct microenvironment‐based subtypes were evaluated between tumor and non‐tumor in TCGA and GEO datasets. CD4/CD8‐T cells were summarized according to CD4/CD8‐central memory, effector memory, and activated‐T cells. (B) Kaplan–Meier analysis of the Tregs immune‐score in the TCGA group. Patients were stratified into high‐(red) and low‐(green) risk groups with maximum cutoff value from survminer algorithm. (C) Subgroup analysis evaluated the prognostic value of the high/low Tregs immune‐score subgroups with different esophageal tumor clinical parameters. The extent of the horizontalline shows the 95% confidence interval for each cohort, and sample size and percentage are shown in the figure. The vertical solid line represents hazard ratio (HR) >1.0 implied that high Tregs‐score is a favorable prognostic biomarker. (D) Distribution of focal and broad copy number alterations in the high/low‐risk subgroups. *ns* represents no statistical difference (*p* > 0.05), * represents statistical difference *p* ≤ 0.05. (E) Gene set analysis barcode plot. The differential gene expression profiles upon Tregs‐relevant risk groups in EC patients (TCGA: Left; GEO: Right) are shown as a shadow bar, where moderated *t* statistic was used for horizontal ranking. In the TCGA datasets, up‐regulated genes upon Tregs‐risk are pink (*t* > 1), and down‐regulated genes are blue (*t* < −1). In the GEO datasets, up‐regulated genes upon EC are pink (*t* > 1), and down‐regulated genes are blue (*t* < −1). The overlap region (gray) is a set of previously described genes in Tregs and macrophages. Red and blue trace lines near the barcode represented relative enrichment. The roast method was used for statistical difference analysis

At the present study, we managed three major steps to establish an accurate and reliable analysis of Tregs‐related immune signatures in EC (Figure 1). To determine whether immune cells in esophageal TME are potential targets for cancer immunotherapy, we firstly evaluated 17 different types of immune cell signature scores between esophageal tumor and non‐tumor groups in the TCGA and GEO datasets. Higher immunosuppressive microenvironmental elements were observed in tumor, including significantly up‐regulated macrophages, MDSCs and Tregs (Student‐*t* test, *p* < 0.05) (Figure 2A, Table S2). Of note, tumor immune cell disorder is a critical step in malignant progression and has been linked to the failure of some clinical tumor immunotherapy.^39^ When taking the patient clinical parameters into account, in TCGA data, we observed that patients with higher Tregs infiltration level were accompanied with shorter overall survival (OS) (Figure 2B). Herein, according to the maximally log‐rank statistics of Tregs immune‐score, we stratified the samples into two distinct groups (high/low) to explore the relationship between Tregs and clinical pathological factors. Within each clinical parameter category, patients were stratified into high and low Tregs signature groups and evaluated Tregs related prognostic risk (Tables S3 and S4). Our results demonstrated that Tregs signatures related poor prognostic risk presented a similar tendency in various malignant progression index, including metastasis, tumor grades and pathological stages (Figure 2C). Due to the sample size, the statistical results are not very significant. Even though, Tregs have the potential to be a predictive factor for pathological stages (HR, III: 1.54, CI_95%_: 0.46–2.61; IV: 1.49, CI_95%_, 0.1–2.89) and tumor grades (G3: HR: 1.3, CI_95%_, 0.32–2.29) in EC patients. Concurrently, Tregs predictive value was also obvious in the tumor development, such as G2 vs G3 (HR: 0.59–1.3), non‐metastasis vs metastasis (HR: 0.64–0.84), and Stage II vs Stage III/IV (HR: 0.86–1.54/1.49). Furthermore, above clinical features and Tregs signatures in the Cox analysis were used to construct the nomogram model, which showed concordance and intuitive predictions (Figure S1A). We found that pathological stages and Tregs signature levels made a considerable contribution to EC patients' poor prognosis prediction compared with other clinical parameters. The nomogram showed a positive predictive tendency, with a C‐index of 0.672. The internal validations with bootstrap approach showed a similar of the C‐index value in the nomogram model and indicated the consistency of prediction. Thus, Tregs might to be one of most important factors in the EC worse clinical outcomes.

Given recently reported evidences, copy number loss is positively associated with immune infiltration.^40^, ^41^ In our study, copy number changes within the different Tregs‐related subgroups were investigated, namely high and low risk cohorts. In terms of somatic copy number aberrations (SCNAs), high‐risk cohort samples presented a higher burden of gain and lower burden of loss (Figure 2D; Table S5), and broad‐level is appeared to be associated with the difference in Tregs‐related immune dysregulation. Moreover, gene‐set profiles showed that global gene expression changes within macrophages and Tregs were associated with EC development and immune dysregulation (Figure 2E). Based on the data from GEO, the expression of selected immune probes also was significantly and highly correlated (*p* < 0.0001) with esophageal tumorigenesis and was accompanied by Tregs/macrophages accumulation (Figure 2E; Right). Similarly, Tregs‐related risk and metagenes expression change occur in the poor prognosis EC patients (TCGA) (Figure 2E; Left). These observations indicated that ongoing specific gene expression dysregulation is critical for immunosuppressive cell‐related risks in EC, such as Tregs and macrophages.

### 3.2 Gene expression changes of tregs in esophageal cancer

**FIGURE 3:**
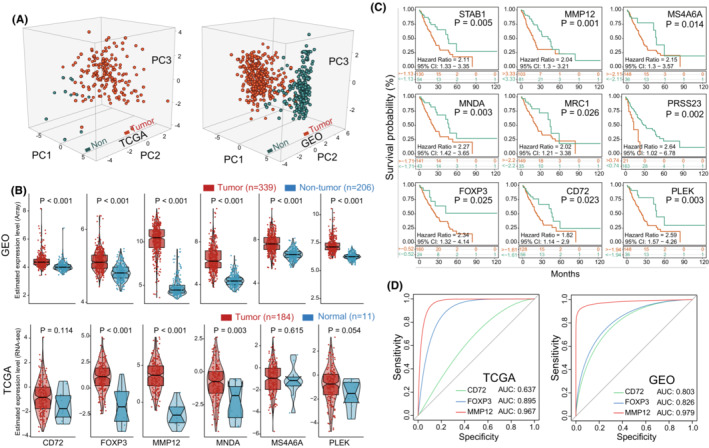
Comparison of Tregs transcriptional variation in esophageal cancer. (A) The scores plot for 3D PCA was based on Tregs‐metagenes expression between EC and non‐tumor from TCGA and GEO datasets. Non‐tumor samples were presented as dark green; tumor samples were presented as orange red. (B) Comparison of Tregs‐metagenes expression between esophageal tumor and non‐tumor in GEO (upper) and TCGA (lower). (C) Kaplan–Meier curves analysis of Tregs‐metagenes in TCGA EC patients. (D) ROC analysis of Tregs‐metagenes expression to predict EC tumorigenesis in the GEO and TCGA datasets

After preprocessing of all EC sample data, we used Tregs‐metagene as a pattern recognition variable. Firstly, principal component analysis (PCA) was applied to distinguish the difference between EC tumor and non‐tumor samples (Figure 3A). PCA is a technique that allows one to visualize relationships and/or difference in complex data sets in standard graphical form.^42^ Among which, we observed a clear separation of Tregs‐related gene set expression between tumor and non‐tumor groups in the TCGA and GEO database (Figure 3A). Then, through comparing the expression in both TCGA datasets and GEO datasets, higher expressed Tregs probe genes were observed in tumor samples compared with normal (Figure 3B) (Partly data not shown). Among which, Foxp3 (*p* < 0.001), MMP12 (*p* < 0.001) and MNDA (*p* < 0.001) were most significantly associated with EC. Furthermore, we analyzed the Tregs probe genes related survival in EC patients, where the result showed similar poor prognosis risk tendency (Figure 3C). Kaplan–Meier curve analysis demonstrated that partly higher‐expressed Tregs probe genes were associated with patients' worse outcome, namely Foxp3 (HR: 2.34, *p* = 0.025), CD72 (HR: 1.82, *p* = 0.023), MMP12 (HR: 2.04, *p* = 0.001), and MNDA (HR: 2.27, *p* = 0.003) (Figure 3C). Finally, by applying receiver operating characteristic (ROC),^38^ we also demonstrated the predictive value of several genes for esophageal cancerization. Our results demonstrated that Foxp3 and MMP12 expression can served as potential predictors for EC in both TCGA (AUC:0.895; AUC:0.967) and GEO (AUC:0.826; AUC:0.979) datasets (Figure 3D; Table S6).

### 3.3 Immune infiltration in esophageal cancer and characteristics of tumor

**FIGURE 4:**
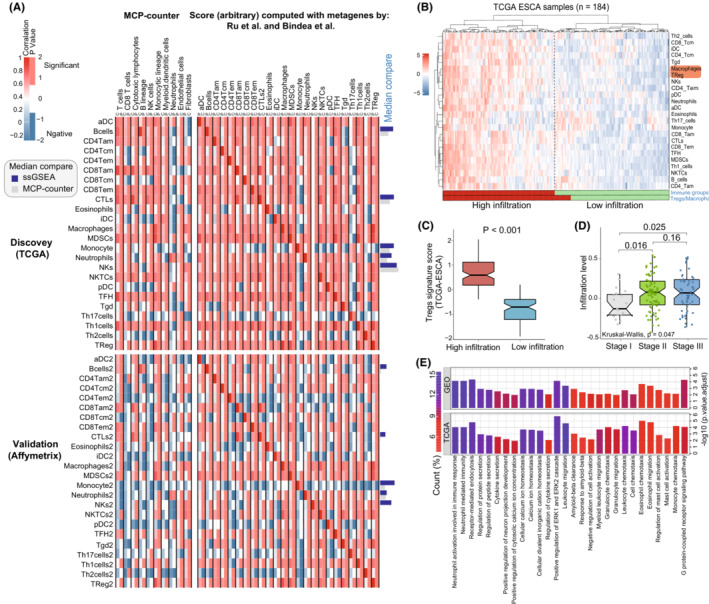
Association between immune cell populations and biological processes. (A) Heatmaps showed infiltration‐scores correlation calculated by immune cells metagene (correlation columns), two sources were used in MCP‐counter and ssGSEA, respectively. The *p* value column indicated the statistical significance of the sample for each immune cell type. The performance of immune cell infiltration relationship was indicated by the concordance between its correlation and the *p*‐ value columns. The right edge bars represented the median prediction scores of identical cells in two methods (navy blue: ssGSEA; gray: MCP‐counter). (B) Unsupervised clustering of 24 types immune cell infiltration for 184 patients in the TCGA dataset. Immuno‐group (high/low‐risk infiltration) are shown with patient annotations. The red/cyan bar represented the immune‐groups clustering according to the 24 immune infiltration cells and Tregs/macrophages. (C) Tregs signature‐score in two groups with different infiltration status. The thick line represents the median value. *p* values was indicated. (D) Distribution of Tregs infiltration score in groups with different clinical pathology (Stage I to III). The Kruskal‐wallis test was used for evaluating the differences among groups (*p* < 0.05). (E) GO enrichment analysis of the Tregs/macrophages related signature genes between EC and non‐tumor. The barplots color depth represented the percentage of genes within each GO term

To further investigate the tumor immune microenvironment of EC, we analyzed the immune‐related features in patients. The relative levels of immune cells infiltration for each EC patient were evaluated in both TCGA and GEO datasets. We systematically assessed the construction scheme of immune cell infiltration and tumor characteristics. To compare the robustness of immune cell parameters in prediction, the different types of immune cell infiltration levels were evaluated by two independent methods, including microenvironment cell populations (MCP)‐counter and the ssGSEA (Established methods) with integrated immune metagenes^31^, ^32^ (Table S7). Both two methods showed high specificity and sensitivity for each corresponding cell population, especially the ssGSEA predictive ability for multiple immune cell subtypes in TCGA (Figure 4A). Not surprisingly, when using the two prediction methods, identical immune cell types from EC patients mostly showed a positive correlation. In terms of prediction value, several immune cells infiltration from MCP‐counter are sometimes negative in the GEO database. Moreover, the ssGSEA confidence prediction vakue (median) was higher than MCP‐counter in both TCGA and GEO datasets.

Given the above observations, unsupervised hierarchical clustering of the EC patients (from TCGA) with matched 24 different immune cells infiltration profile was performed, and the results were presented in Figure 4B. A strong correlation between the immunosuppression elements Tregs and macrophages in the high immune‐infiltration group was observed (Figure 4B). To further investigate the role of Tregs in TME, we evaluated Tregs signature‐score between the high and low infiltration group. We observed that high immune infiltration was positively correlated with higher Tregs signature‐score (Figure 4C). Moreover, higher level of Tregs and macrophages related immune infiltration were significantly associated with tumor progressionion, especially the pathological stage I to III (Figure 4D). Recent study demonstrated that crosstalk between Tregs and macrophages activation contributes to potential immunosuppression in the TME, where Tregs show a moderation effect on macrophages.^43^ In this aspect, our results supported that Tregs might be involved in various tumor regulation mechanisms of EC patients. To demonstrate the underlying biological behavior of Tregs‐related pattern, we analyzed the Tregs and macrophages metagenes altered expression by limma package. Moreover, ClusterProfiler was applied to execute GO enrichment analysis for these gene biological functions. In both TCGA and GEO datasets, these altered genes expression has significant influence on the type of biological functions like immune cells migration, activation and receptor functions, which is remarkably related to immune cells regulation and immunity processes in EC development (Figure 4E; Table S8). Taken together, the results confirmed again that Tregs‐related function plays a non‐negligible role in the modulation of EC immune microenvironment.

### 3.4 Tregs related STAT3 and transcriptome traits Characteristics

**FIGURE 5:**
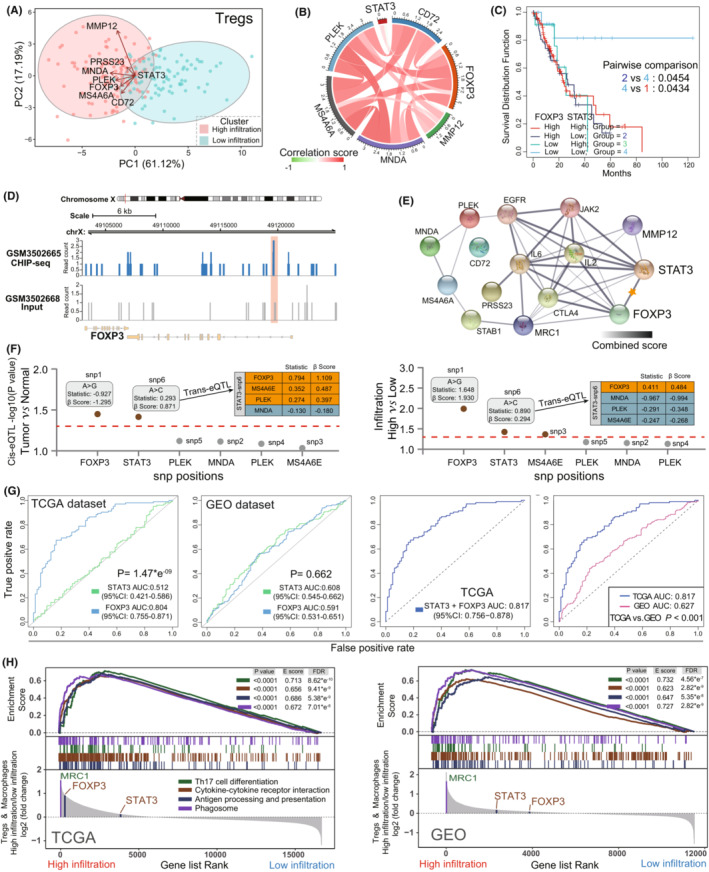
STAT3 function in Tregs transcriptional traits characteristics. (A) Principal component analysis (PCA) score plots for the Trges‐metagene transcriptome profiles in different immune infiltration cohorts of TCGA dataset. The remarkable difference on transcriptome was distinguished between different infiltration patterns. (B) Correlation analysis between STAT3 and Tregs‐metagenes in EC. (C) Survival analysis for Foxp3 and STAT3 subgroup patients by using Kaplan–Meier curves. *p* values were indicated in the graphs. (D) STAT3 binding at Foxp3 loci in naïve B cells was determined by ChIP‐Seq. The *y* axis indicates the number of mapped sequencing reads. Input control showed background STAT3 binding (lower; gray bars). (E) Protein–protein interaction (PPI) network for the Tregs‐relevant proteins and STAT3. Thicker lines and deeper color represented the closer relationship to each other. (F) Cis‐eQTLs and trans‐eQTLs for STAT3 and Foxp3 in two subgroups. Left is the comparison between tumor and normal, right is the comparison between Tregs/macrophages related high infiltration and low infiltration, positive trans‐eQTLs results were marked in color. Significant novel SNPs from cis‐eQTL are indicated by the brown annulus. The statistics used is correlation coefficient “r”; and β‐Score is the confidence index. The red line corresponds with a threshold of *p* ≤ 0.05. (G) ROC curves measured the predictive value of the Foxp3, STAT3 (Left), as well as combination of Foxp3 and STAT3 (Right) in the Tregs‐relevant infiltration. Comparison of ROC curves for combined‐STAT3/Foxp3 were used to evaluate the predictive ability of Tregs infiltration in TCGA and GEO EC patients (Right). (H) Enrichment plots showed Th17 cell differentiation, cytokine‐cytokine receptor interaction, antigen processing and presentation in the high infiltration groups. Each run was performed with 1000 permutations. *p*‐values were determined by the Kolmogorov–Smirnov test

To explore Tregs underlying regulation mechanisms in the immune infiltration of EC, a comprehensive transcriptional analysis was generated to explore association between transcriptional regulatory factor STAT3 and Tregs specific gene. We performed PCA plot analysis according to the obtained Tregs‐metagene and STAT3 in the different infiltration groups. There was significant distinction existed in the Tregs metagene and STAT3 transcriptional profiles between the high‐ and low‐infiltration groups in TCGA (Figure 5A). Through pearson correlation analysis, as shown in Figure 5B, STAT3 was observed to be positively associated with Foxp3 expression in EC patients (*R*:0.225). Foxp3 is a key modulator for Tregs specific recognition and immune suppressive function,^44^ while its relationship to STAT3 is still unclear in EC. At the present study, we will try to investigate the association between STAT3 and Foxp3 in EC. Indeed, we found that EC patients with *STAT3* ^low^ *Foxp3* ^low^ showed a longer survival than the other subgroups (Log‐rank *p* value<0.05) (Figure 5C). Then we carried out a visual genome inspection of aligned sequence reads by CHIP‐seq from GEO database. As an auxiliary evidence, representative genome browser of STAT3 read peak in comparison with input peak (control) in naïve B immune cell are shown in Figure 5D, which is obviously enriched in part of Foxp3 gene regions. In consistent with this, the protein–protein interaction (PPI) network analysis from STRING^45^ confirmed the functional attributes of STAT3 on the Tregs marker gene Foxp3 (Figure 5E; Table S9). Meanwhile, we noticed a significant increase of STAT3 and Foxp3 expression in EC and higher Tregs/macrophages related infiltration groups, which was associated with single‐nucleotide polymorphism (SNP) (Figure 5F). The cis‐expression quantitative trait locus (cis‐eQTLs) analysis results showed that genomic SNP was positively associated with two signatures expression in EC (STAT3: statistics r: 0.293, β‐Score: 0.871) and Tregs/macrophages‐related infiltration (STAT3: statistics r: 0.890, β‐Score: 0.294; Foxp3: statistics r: 1.648, β‐Score: 1.930). Moreover, in terms of SNPs distant effect, trans‐eQTL results indicated that STAT3 expression was positively associated with Foxp3 expression in both tumor (statistics r: 0.794, β‐Score: 1.109) and higher infiltration groups (statistics r: 0.411, β‐Score: 0.484). Therefore, we speculated that STAT3 regulation functions was potentially associated with Tregs Foxp3 expression change and influenced the Tregs infiltration ability in EC patients.

In the current results, STAT3 did not present a predictive advantage in Tregs infiltration when compared to Foxp3 (TCGA, likelihood ratio test, *p*: 1.47*e^−9^, Figure 4G‐Left). While, combining STAT3 and Foxp3 showed a better predictive value than STAT3 or Foxp3 alone (AUC: 0.817 vs. 0.804/0.512) (Figure 4G‐Right; Table S6). Meanwhile, in GEO dataset, there are similar predictive ability to Tregs infiltration between STAT3 (AUC: 0.608) and Fxop3 (AUC: 0.591). We further validated the diagnostic performance of established TCGA in another GEO validation set. The AUC value of combined STAT3 and Foxp3 in TCGA was 0.817 (CI_95%_: 0.756–0.878), which was significant higher than GEO cohorts (AUC: 0.627, CI_95%_: 0.568–0.687) (likelihood ratio test, *p* < 0.001) (Figure 4G‐Right). To further explore the Tregs function in TME of EC, in both TCGA and GEO datasets, based on the GSEA analysis, we ranked Tregs and macrophage transcripts with log2 (Fold Change) between high and low infiltration groups (Figure 4H; Table S10). We observed that the enriched expression of these probes was considered as immune cell differentiation and activation^46^, ^47^ in high infiltration group. Taken together, our results suggested that Tregs infiltration is closely associated with risk of EC, including poor prognosis and tumor development. Simultaneously, STAT3/Foxp3 axis potentially enforced the Tregs function in TME of EC and served as a promising target for the anti‐tumor immunotherapy.

## 4 DISCUSSION

Tregs are genetically and functionally linked to the pathogenesis of human tumors,^48^, ^49^, ^50^ including gastrointestinal tumors.^19^ However, our understanding of Tregs function and underlying mechanisms in EC are still limited.^51^, ^52^ In this comprehensive study, we observed that Foxp3 related Tregs signature and infiltration with its intrinsic metagenes were essential for EC and systemic immunosuppression functions. Higher Tregs level in EC patients contributed to poor prognosis and tumor development. Furthermore, STAT3 and Foxp3 interaction takes part in Tregs‐related immunosuppression in various ways. Firstly, STAT3/Foxp3 axis was associated with tumor progress in EC and enhanced Tregs infiltration, as well as the potential treatment option for Tregs inhibition. Moreover, STAT3/Foxp3 axis related signaling cascades were crucial for Tregs immunosuppression functions in TME of EC. Through combining multiple methodologies, we speculated that STAT3/Foxp3 cascade is a potential target for Tregs and associated with EC development, which have not been reported previous. Discriminating the different Tregs regulation patterns in the immune microenvironment potentially promote our understanding of the antitumor immune response in EC, which is also helpful to effective immunotherapy guidance.

Immunosuppressive cells have the ability to impair the microenvironment of various tumor foci,^53^ which has been found to be connected with tumorigenesis, development and metastasis,^54^, ^55^ including EC.^56^, ^57^ Tumor‐infiltrating lymphocytes (TILs) is an intricate concept that makes it difficult for immune cells to better compete with tumors.^58^, ^59^ Several studies have reported that specific types of immune cell regulation can release anti‐tumor effector immune responses in EC.^56^, ^60^ Therefore, biomarker prediction is valuable for immune cells immunotherapy, which is utmost importance in ameliorating the therapeutic benefit.^60^ Here, we provided a direct evidence that dysregulation of various types of immune cell between tumor and non‐tumor was associated with EC patients, including down‐regulated immune activation cells (CD4^+^/CD8^+^ T_cells and DCs) and upregulated immunosuppression cells (macrophages, MDSCs and Tregs). Current studies demonstrated that immunosuppression cells in TME have the ability to promote EC progression^56^, ^61^ and diminish conventional therapy (radiotherapy and chemotherapy) effect^62^ through various signaling pathways. In our study, we observed that immunosuppression characterized by Tregs was an independent prognostic factor for EC. Tumor‐related Foxp3^+^ Tregs were frequently detected in residual metastasis and primary tumor foci, while the decreased Foxp3^+^ Tregs were supposed to be associated with pathological remission and therapy effects.^63^, ^64^, ^65^ Moreover, tumor‐derived CD4^+^CD25^+^ Tregs activation was reported to inhibit the maturation and function of the DCs, which will abolish antigen‐specific immune responses and promote the tumor progression.^66^ In EC, the accumulation of CD4^+^CD25^+^‐ and Foxp3^+^‐Tregs was supposed to be connect with cancer cell proliferation and poor clinical prognosis.^52^, ^67^

In the present study, different computational algorithms and Tregs related gene signatures were used to investigate the Tregs dysregulation in EC. Based on two cohorts from different sources, potential biomarkers expression pattern of Tregs in EC patients were comprehensively depicted at the transcriptional level. Our results showed that Foxp3 related Tregs were significantly upregulated in EC, which also showed a close relationship to patients' clinical outcomes. Higher Tregs signature level is closely associated with EC development, such as increasing the pathological stages, tumor grades and metastasis related prognosis risk. Nomograms is an accurate tool for exploring relevant indicators that affect the outcome in multifactor regression analyses and are commonly used for many cancers clinical prognosis prediction.^68^, ^69^ By which, we observed a similar result that Tregs signature levels were positively related to EC patients' poor prognostic risk. Therefore, Foxp3‐related Tregs potentially play an important role in EC development. Moreover, Cui et al^52^ identified the role of Foxp3^+^ Tregs in EC by immunohistochemistry analysis, where they revealed that Tregs biomarker expression was greatly associated with Tregs accumulation in EC patients. Among which, IL‐33 recognizes the receptor ST2 in Foxp3^+^ Tregs, then promoting the Foxp3^+^ Tregs accumulation and immunosuppressive functions in tumor. In our study, Tregs‐metagene expression pattern was analyzed across nearly five hundred EC patients from two large independent cohorts. In line with previous study,^52^ we observed that Tregs probe genes expression in tumor was significantly different from non‐tumor, which was also associated with patient poor prognosis, especially Foxp3 and MMP12. Generally, Tregs‐specific Foxp3 is indispensable for immune system homeostasis and protects Tregs identity in response to abnormal signals,^70^ whereas elevated Foxp3^+^ Tregs level showed a negative correlation with prognosis in various tumor types.^50^ Although there was no significant statistical difference in multivariate analysis, higher Foxp3^+^ Tregs signature was observed to be associated with worse EC patient's clinical outcomes, such as metastasis, pathological and tumor grade development. Infiltration of Foxp3^+^ Tregs potentially induced immunosuppression and stimulated macrophages to promote the development of EC.^43^ Crosstalk between Foxp3^+^ Tregs and macrophages responsible for the failure of immuno‐surveillance and specific targeting of related cascades may reactivate anti‐tumor immunity in EC.^43^ In addition, previous study observed that Tregs‐specific MMP12 was involved in the recruitment of neutrophils or facilitating their clearance,^71^ which is unclear in EC. Peng et al^72^ revealed that accumulation of MMP12 in the microenvironment also promoted Tregs proliferation and immunosuppression in tumorigenesis. On the other hand, we also observed that genomic instability was partly associated with Tregs‐related risk, higher Tregs level group with genetic alteration were mostly correlated with Tregs prob genes upregulation. Of note, higher expression of Tregs‐specific genes is a good predictor for immunosuppressive effects and deterioration in tumors, especially Foxp3.^73^ In the future, it will be important to further examine how the complex Tregs intrinsic molecules regulation promote immunosuppressive functions in EC patients.

Integrated analysis revealed that there was strong evidence between immune metagenes transcription and immune cell infiltration. We screen GEO and TCGA datasets by two computational methods with rigorous, unbiased, and conservative approach to measure signals in gene expression. In line with previous interpretations, MCP‐counter scores corresponded to the cell population abundance across multiple samples, while it usually presents absolute abundance at relatively low frequencies in the samples.^27^ In contrast, ssGSEA aimed at estimating the relative proportion of immune cell population within a single sample.^32^ Both established methods depicted the positive immune infiltration and correlation between different immune cells in EC. Moreover, we observed a stronger infiltration signal from TCGA dataset in two independent methods, which corresponded with above findings. Interestingly, we noticed that infiltration levels depended on the gene expression were a little bit obvious in ssGSEA compared to MCP‐counter, suggesting that ssGSEA has a higher predictive value to evaluate TME for further advance personalized immunotherapy in EC. We also observed that Tregs infiltration was positively correlated with macrophages, which have been confirmed to be closely associated with tumorigenesis and immunosuppressive microenvironment,^74^, ^75^, ^76^ including EC.^77^ With the ligand–receptor interaction, like HLA and LILRB1, Foxp3^+^ Tregs showed the ability to influence macrophages and contribute to effector T cells exhaustion in EC.^43^ Furthermore, our data indicated that patients with the higher infiltration of immunosuppressive cell Tregs exhibited worse clinical outcome, namely tumor pathological stages. Consistent with the previous studies^78^, ^79^, ^80^ emphasized that Tregs activation and accumulation are core mechanisms of tumor progression, where intense infiltration of Foxp3^+−^Tregs was reported to contribute to shorter survival time and 14‐fold rate of tumor recurrence compared to negative cases. On the other hand, Tregs are necessary for self‐tolerance and autoimmune defense, and the deficiency of Tregs will lead to lethal autoimmunity disease.^81^, ^82^ The absence of Foxp3^+^ Tregs was involved in some autoimmune disease, like immune polyendocrinopathy, enteropathy, pemphigus.^83^, ^84^ Thus, monitoring and keeping Tregs balance can ameliorate functional immunity and disease processes, as well as improving the cancer treatment effect. This resource potentially helps us to improve the effects of personalized immunotherapy and decipher the different immune interplay.

Equally importantly, we provide evidence that STAT3 might take part in the Foxp3 expression by regulating transcript modifications or other fields. This adds another layer of complexity to STAT3 role in Tregs as a transcriptional regulator. In terms of this, STAT3 and Foxp3 interaction site was exist in the promoter of tumor induced Tregs and associated with several tumors progression.^26^, ^85^ The STAT3/Foxp3 axis suppression in Tregs from head and neck squamous cell carcinoma contribute to Treg reprogramming and activation of antigen‐presenting cells, ultimately improved head and neck tumor growth delay.^86^ Moreover, inhibiting the IL‐6‐STAT3/Foxp3 cascade showed immune‐related antitumor effect in hepatocellular carcinoma, wherein IL‐6‐STAT3 signaling inactivation suppressed Foxp3 expression in Tregs.^87^ However, in colorectal cancer patients inflammatory infiltrate response, the the upregulate of STAT was negatively associated with Foxp3+ T lymphocytes and potential to relieve local and systemic inflammation.^88^ These studies underscore the complex interplay between the STAT3/Foxp3 axis and Tregs and carry significant implications for drug targeting and immune TME modulation combination approaches in various tumor immunotherapy. We showed the distinct expression patterns of Tregs marker genes between high‐/low‐risk infiltration groups. Previous study demonstrated that Tregs Foxp3 expression level was directly connected to the level of STAT3 in skeletal immune system and breast cancer,^24^, ^89^ and STAT3 activity is also correlated with poor prognosis in EC patients who had undergone surgical resection or chemoradiotherapy.^6^, ^90^, ^91^ Generally, according to the stability of the genome, small‐scale geographical differences will not affect the accuracy of the results. Based on the gene expression, ChIP‐seq, eQTLs and PPI network analyses, we speculated that direct crosstalk between STAT3 and Foxp3 potentially influenced the EC patients' prognosis and participate in Tregs related immune regulation. Recently, SNPs' locus in cancer have been demonstrated to influence the immune checkpoint related immune disorder and target gene expression.^92^ According to the transcriptional regulation analysis of eQTLs, the cis‐eQTLs results indicated that SNPs in EC have a significant influence in STAT3 expression. Meanwhile, the trans‐eQTLs demonstrated that distant function of STAT3 to Foxp3 expression was also important in EC progress and Tregs/macrophages‐related immunosuppression. As previous study reported that STAT3 binding site of first intron of the Foxp3 gene was involved in Foxp3 expression in tumor Tregs, such as renal cell cancer, lung cancer, melanoma, colon cancer, and rectal cancer.^93^ Through some specific receptors activation, the STAT3/Foxp3 axis will be stimulated and accompany with Foxp3 and cytokine upregulation in Tregs, which directly influence the antitumor immunity.^94^ According to the functional analysis of Tregs/macrophages‐relevant markers, our results demonstrated that several immunomodulatory functions were associated with Tregs infiltration, including Th17 cells differentiation, cytokine‐cytokine receptor interaction, and antigen processing and presentation. Together, these findings support the specific role that combined STAT3 and Foxp3 could be a more predictive biomarker for Tregs infiltration than STAT3 or Foxp3 alone.

There are several limitations in our study. More prospective cohort of clinical EC samples with different Tregs status and STAT3/Foxp3 expression is supposed to conquer the limitation of data. This study used some methods to verify the interaction between immune cell disorder and esophageal cancer clinical features. The robustness and clinical usefulness of these approaches need further external validations in clinical trials, such as immune cell prediction and nomogram evaluation. In addition, gene expression profiles to sampling bias due to different sources and individuals, which can be adjusted by optimized algorithms and eligible samples in the future. However, in‐vitro and in‐vivo experimental validation is still an important step to further verify the prognostic value of the Tregs signature genes and relevant tumor microenvironment. And the lack of experimental verification is the limitation of this study. In this study, not all patients are affected by the only biological function of Tregs, and more immune microenvironment features should be incorporated into consideration for improving accuracy in the future.

## 5 CONCLUSION

In conclusion, our results observed the important of Tregs functions and its potential regulation mechanisms in EC, especially the crosstalk between STAT3 and Foxp3, which has not been clarified before. The differences in Tregs infiltrations and related biological processes that should not be ignored, which potentially cause the tumor heterogeneity, poor prognosis and complex immune microenvironment. In this study, comprehensive landscape of the cellular, molecular, and genomic characteristics associated Tregs infiltration patterns yielded several important insights that shed light on how Tregs dysregulated in EC. These observations may be applied to expand the understanding of EC patients' anti‐tumor immunotherapy.

## AUTHOR CONTRIBUTIONS

Y.L. and Z.Q.J. conceived and drafted the manuscript. Y.L. revised the manuscript. Z.J. and L.Y. provided administrative and technical support. W.X. and C.P. participated in data organization. R.J.F. and J.J. gave the concepts of the manuscript. R.J.F. and J.J. approved the version to be submitted. All authors contributed to the article and approved the submitted version.

## FUNDING INFORMATION

Not applicable.

## CONFLICT OF INTEREST

The authors declare no conflicts of interest.

## ETHICAL APPROVE STATEMENT

Institutional review board approval was obtained from the The second people's hospital of yibin to analyze The Cancer Genome Atlas and Gene Expression Omnibus records data.

## Supporting information


Figure S1

Tables S1‐S10

## Data Availability

All the original data of the study are available from the corresponding author on reasonable request. All data are available from public database and the source data for the figures have been uploaded in the supplementary tables.
