# Supplementary figures and images for: Investigation on the regulatory T cells signature and relevant Foxp3/STAT3 axis in esophageal cancer
Source: Cancer Med. 2022 Oct 13;12(4):4993–5008. doi: 10.1002/cam4.5194 (PMC9972178; doi:10.1002/cam4.5194)

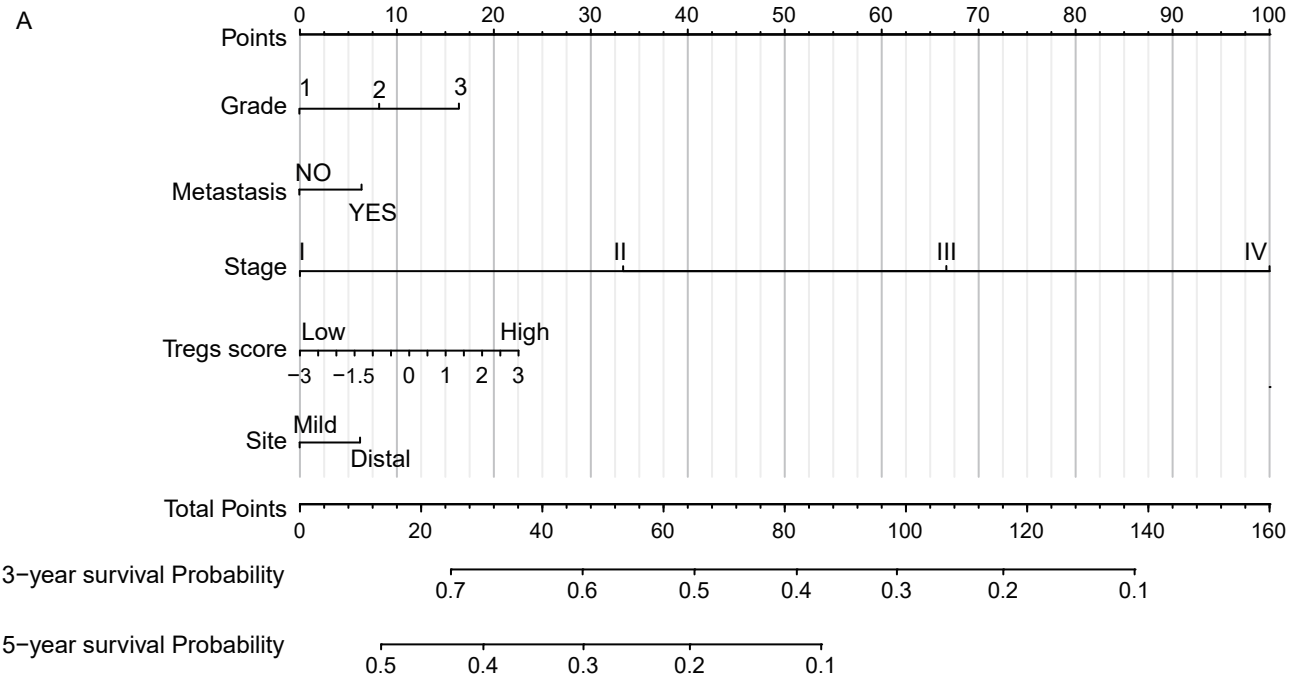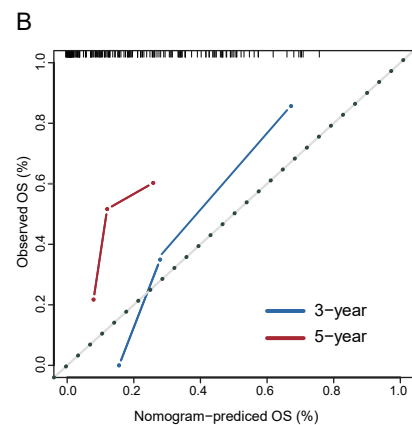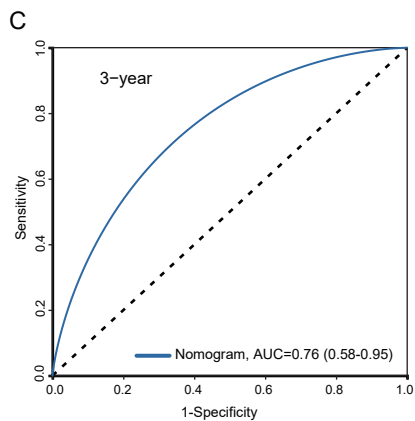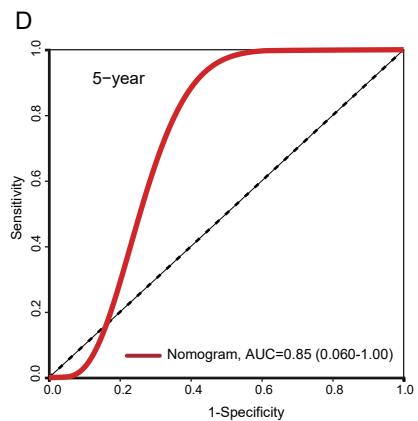

Supplement: Supplementary file 1 — Figure S1 [file CAM4-12-4993-s001.pdf]
